# All-*trans* Retinoic Acid and Beta-Carotene Increase Sclerostin Production in C2C12 Myotubes

**DOI:** 10.3390/biomedicines11051432

**Published:** 2023-05-12

**Authors:** Franz Ewendt, Anne Lehmann, Maximilian F. Wodak, Gabriele I. Stangl

**Affiliations:** 1 Institute of Agricultural and Nutritional Sciences, Martin Luther University Halle-Wittenberg, 06120 Halle, Germany; 2 NutriCARD Competence Cluster for Nutrition and Cardiovascular Health, Dornburger Str. 25, 07743 Jena, Germany

**Keywords:** vitamin A, carotenoids, retinoids, myokine, retinoic acid receptor, bone growth, muscle–bone crosstalk

## Abstract

Sclerostin is a protein secreted by osteocytes whose encoding gene *SOST* is regulated by mechanical stimuli, cytokines, and all-*trans* retinoic acid (ATRA) and mediates antianabolic effects on bone formation as an inhibitor of the canonical Wnt/β-catenin pathway. Interestingly, skeletal muscle has recently been identified as another source of sclerostin, suggesting that the musculature may play an important role in maintaining bone mass. However, regulators of muscular *SOST* expression are virtually unknown. This study investigates the influence of ATRA and the provitamin A derivative beta-carotene (β-C) on sclerostin synthesis in muscle cells. The impact of ATRA, its synthetic analog TTNPB, and β-C on *Sost* transcription was analyzed by qRT-PCR in C2C12 myotubes and the secreted sclerostin protein by ELISA. ATRA strongly increases the sclerostin synthesis in C2C12 myotubes in a dose-dependent manner. The stimulating effect of ATRA and TTNPB on *Sost* is largely reduced in the presence of the retinoic acid receptor inhibitor AGN193109. β-C also increases the *Sost* expression, but this effect vanishes when β-C is coincubated with beta-carotene 15,15′-monooxygenase 1 (BCMO1)-specific siRNA. Thus, ATRA is a potent stimulator of sclerostin release in muscle cells. β-C can also increase *Sost* mRNA abundance, but this effect depends on the conversion to a retinoid.

## 1. Introduction

Skeletal muscles and bones are anatomically and biochemically linked and play critical roles in locomotion and metabolism in humans [1]. The interaction of bone and muscle has attracted increasing interest, as the two tissues are not only physically linked but can influence both muscle and bone metabolism, as well as the homeostasis of distant tissues such as the heart, liver, immune system, or adipose tissue through the release of biochemical signals [2,3,4,5,6]. These signals are called myokines if they are secreted by muscle cells or osteokines if they are produced by bone cells [7]. Secreted myokines and osteokines can exert autocrine and paracrine effects and, after entering the circulation, endocrine effects in surrounding or distant cells and tissues [1,7]. The production and release of myokines and osteokines are prerequisites for the molecular communication between muscles and bones, which has recently been termed muscle–bone crosstalk [3,7]. 

Myostatin was identified as the first secreted myokine which binds to autocrine receptors and inhibits muscle growth [8] but also exerts endocrine functions by inducing osteoclast differentiation [9] or increasing the formation and secretion of fibroblast growth factor 23 (FGF23), a phosphaturic osteokine, in bone cells [10]. Since the discovery of myostatin, an increasing number of myokines, such as interleukin-6, -7, and -15, insulin-like growth factor 1, brain-derived neurotrophic factor, FGF21, irisin, and decorin, have been identified to be secreted by skeletal muscles, and, after entering the blood, these molecules mediate endocrine effects on bone and other tissues [6,11]. However, the responsible regulators and underlying mechanisms controlling the formation and release of these signaling molecules have not been fully identified. Notably, sclerostin, originally identified as an osteokine secreted by bone cells [12], was recently identified as a novel myokine through its expression and secretion by muscle cells [13]. The fact that sclerostin is formed and secreted in both bone and muscle demonstrates the mutual importance of both tissues for each other. Sclerostin, a 24-kDa protein, is encoded by the *SOST* gene and is mainly synthesized and secreted in bone tissues by osteocytes and osteoblasts [12,14]. Sclerostin has an antianabolic effect on bone because it acts as an antagonist of the canonical Wnt/β-catenin pathway [15,16], resulting in decreased bone mass and bone growth, decreased formation of bone matrix by osteoblasts, inhibition of osteoblast proliferation and differentiation into osteocytes, and indirect promotion of bone resorption [17]. Loss-of-function mutations of the human *SOST* gene cause sclerosteosis, which has a phenotype similar to that of rare hereditary van Buchem disease, which is characterized by excessive production of bone mass [12,18,19]. Additionally, sclerostin is associated with liver diseases, vascular calcification, osteoarthritis, and cancer [20]. The transcription of *SOST* in bone cells is mainly regulated by mechanical stimulation but also by various hormones such as parathyroid hormone, 1,25(OH)_2_D_3_ (1,25-dihydroxyvitamin D, calcitriol), and estrogen or cytokines such as interleukin-1β, tumor necrosis factor α, and transforming growth factor β or bioactive metabolites and endocrine factors such as calcitonin, bone morphogenetic protein, prostaglandin E_2_, and retinoic acid, the active metabolite of vitamin A [21]. Vitamin A (retinol) is taken up by enterocytes as retinyl esters from foods such as eggs, liver, or milk or as provitamin A, particularly beta-carotene (β-C), from vegetables such as carrots or spinach. Ingested (pro)vitamin A is then incorporated into chylomicrons, transported into the bloodstream and taken up and mainly stored by hepatocytes [22]. Other tissues that take up relevant quantities of food-derived retinoids are bone [23] and skeletal muscle [24]. Once in the target cells, retinol is first oxidized by alcohol dehydrogenases (ADH) to all-*trans* retinal and, in a further step, oxidized by retinal dehydrogenase (RALDH) to the metabolic active form, all-*trans* retinoic acid (ATRA) [22]. The carotenoid β-C can also be processed into retinal by the enzyme beta-carotene 15,15′-monooxygenase (BCMO1) [25], thus serving as an alternative precursor for cellular ATRA production [26]. ATRA mediates its biological effects by entering the nucleus, where it binds to the retinoic acid receptor (RAR). The RAR then dimerizes with the retinoid X receptor (RXR) and binds to retinoic acid response elements (RAREs) in the promoter regions of target genes, thus inducing the transcription of ATRA-regulated genes [22]. ATRA has a crucial role in bone and muscle tissue homeostasis and function [22,27,28,29,30,31], and interestingly, increasing evidence has identified a regulatory role in myokine and osteokine production [32,33,34,35]. 

Although muscle cells are capable of producing sclerostin, little is known about potential regulators. This study investigates the role of ATRA and β-C in the regulation of sclerostin production in C2C12 myotubes.

## 2. Material & Methods

### 2.1. Cell Culture and Treatments

Murine C2C12 muscle myoblasts (CRL-1772; ATCC, Manassas, VA, USA) were cultured under proliferative conditions at 37 °C and 5% CO_2_ in a growth medium containing Dulbecco’s modified Eagle’s medium (DMEM) with 4.5 g/L D-glucose, L-glutamine, and no pyruvate (Gibco, Life Technologies, Darmstadt, Germany) supplemented with 10% fetal bovine serum (FBS), 100 U/mL penicillin, and 100 µg/mL streptomycin (PenStrep; both FBS and PenStrep from Gibco, Life Technologies, Darmstadt, Germany). C2C12 cells for treatment studies were used between the 10th and 20th in-house passages. For experiments, 1 × 10^5^ cells were seeded per well on 6-well plates and cultured in the growth medium for 48 h. The differentiation of C2C12 myoblasts into C2C12 myotubes was initiated after 48 h of proliferation, when the wells were 80–90% confluent with cells. For this, the growth medium was replaced by a differentiation medium containing DMEM with 4.5 g/L D-glucose and L-glutamine without pyruvate supplemented with 50 U/mL penicillin, 50 µg/mL streptomycin (both DMEM and PenStrep from Gibco, Life Technologies, Darmstadt, Germany), and 2% horse serum (Sigma–Aldrich, Schnelldorf, Germany). At day 3 of differentiation, the formed C2C12 myotubes were treated with ATRA (1–10^4^ nM) or the retinoic acid analog and RAR agonist TTNPB (4-[(E)-2-(5,6,7,8-tetrahydro-5,5,8,8-tetramethyl-2-naphthalenyl)-1-propenyl]benzoic acid; 1–10^4^ nM) (both from Tocris, Bristol, UK) or β-C (100–400 nM; Sigma–Aldrich, Schnelldorf, Germany) for 24 h. In some experiments, C2C12 myotubes were pre-stimulated with or without the pan-RAR antagonist AGN193109 (4-[2-[5,6-dihydro-5,5-dimethyl-8-(4-methylphenyl)-2-naphthalenyl]ethynyl]benzoic acid; 1 µM; Tocris, Bristol, UK) 3 h before being treated for an additional 24 h with ATRA, TTNPB (both 100 nM), or β-C (400 nM).

### 2.2. Silencing

For the silencing of *Bcmo1*, 1 × 10^5^ C2C12 myoblasts were seeded per well for 48 h in growth medium. After 48 h of proliferation, the growth medium was removed, and the cells were washed with PBS and then transfected in antibiotic-free differentiation medium using 100 nM ON-TARGETplus Mouse SMARTpool *Bcmo1* siRNA (L-057708-01-0010) or 100 nM ON-TARGETplus nontargeting control siRNA (D-001810-10-20) and 7.5 µL of DharmaFECT 1 transfection reagent (all reagents from Dharmacon, Lafayette, CO, USA). After 72 h of *Bcmo1* silencing, the siRNA-containing antibiotic-free differentiation medium was renewed. After an additional 24 h of silencing, the C2C12 myotubes were incubated with 400 nM β-C or with vehicle alone in the presence or absence of *Bcmo1*-specific or nontargeting siRNA for another additional 24 h. In the C2C12 myotubes treated with *Bcmo1*-specific siRNA, the relative *Bcmo1* expression was 1.72 × 10^−6^ ± 4.5 × 10^−7^ arbitrary units and 1.70 × 10^−5^ ± 2.29 × 10^−6^ in the control cells treated with nontargeting siRNA (*p* < 0.001). 

### 2.3. Qualitative Expression Analysis

Untreated murine C2C12 myotubes were used for the total RNA extraction with TriFast reagent (Peqlab, Erlangen, Germany). The cDNA was synthesized at 25 °C for 5 min, 42 °C for 1 h, and 70 °C for 15 min using 1.2 µg of total RNA, random primers, and the GoScript^TM^ Reverse Transcription System (both from Promega, Mannheim, Germany). For cDNA amplification, an RT–PCR was performed using a Rotor-Gene Q Cycler (Qiagen, Hilden, Germany) with 2 µL of the synthesized cDNA and the following program: 95 °C for 3 min and 40 cycles of 95 °C for 10 s, 59 °C for 30 s, and 72 °C for 30 s. Amplified RT–PCR products were loaded on a 1.5% agarose gel and visualized by Midori Green (Biozym, Hessisch Oldendorf, Germany). The following primers (5′ → 3′ orientation) were used:

Mouse *Bcmo1*: 

F: CCCTCGGATAAATTATGCTTAC

R: GGACATCATCTTCATCCTTC

### 2.4. RNA-Isolation and Quantitative Real-Time PCR

The total RNA was extracted from the murine C2C12 myotubes using a TriFast reagent (Peqlab, Erlangen, Germany) according to the manufacturer’s protocol. Next, 1.2 µg of total RNA was used for cDNA synthesis (25 °C for 5 min, 42 °C for 1 h, and 70 °C for 15 min) with the GoScript^TM^ Reverse Transcription System and random primers (both Promega, Mannheim, Germany). For the determination of relative *Sost* and *Gapdh* expression, a quantitative reverse-transcription real-time PCR (qRT–PCR) was performed using a Rotor-Gene Q Cycler (Qiagen, Hilden, Germany) and GoTaq qPCR Master Mix (Promega, Mannheim, Germany) under the following conditions: *Sost*: 95 °C for 3 min and 40 cycles of 95 °C for 10 s, 59 °C for 30 s, and 72 °C for 30 s; *Gapdh*: 95 °C for 3 min and 20 cycles of 95 °C for 10 s, 58 °C for 30 s, and 72 °C for 30 s. The calculated relative mRNA transcript level of *Sost* was normalized to the expression level of *Gapdh* in the same cDNA sample. The quantification of *Sost* gene expression is presented as 2^−ΔCT^ (ΔC_T_ = C_T_ [target gene] − C_T_ [reference gene]) transformed data [36]. The following primers (5′ → 3′ orientation) were used: 

Mouse *Sost*:

F: TCAGGAATGATGCCACAG

R: GTACTCGGACACATCTTTG

Mouse *Gapdh*:

F: GGTGAAGGTCGGTGTGAACG

R: CTCGCTCCTGGAAGATGGTG

### 2.5. Enzyme-Linked Immunosorbent Assay (ELISA)

C2C12 myoblasts were cultured and differentiated into myotubes as described above and treated at day 3 of differentiation with 10 nM ATRA, 100 nM ATRA or vehicle alone for another 24 h. Next, the cell culture supernatant was collected and stored at −80 °C. For the quantification, cell culture supernatants were concentrated using Vivaspin 6 centrifugal concentrators (Sartorius, Göttingen, Germany), and the sclerostin protein was subsequently determined using an ELISA kit (Mouse/Rat SOST, R&D Systems, Inc., Minneapolis, MN, USA) according to the manufacturer’s protocol. 

### 2.6. Statistics

The data are shown as arithmetic means ± SEM; *n* represents the number of independent experiments. Data were tested for normal distribution using the Shapiro-Wilk normality test. Comparisons of more than two treatments were tested for significant differences with one-way ANOVA followed by Dunnett’s or Tukey’s multiple comparison (if necessary, with Welch’s ANOVA followed by Dunnett’s T3 multiple comparison test) or the Kruskal–Wallis test followed by Dunn’s multiple comparison test for non-normally distributed data. Differences were considered significant at *p* < 0.05. 

## 3. Results

### 3.1. All-trans Retinoic Acid Stimulates Sclerostin Production in C2C12 Myotubes

**Figure 1:**
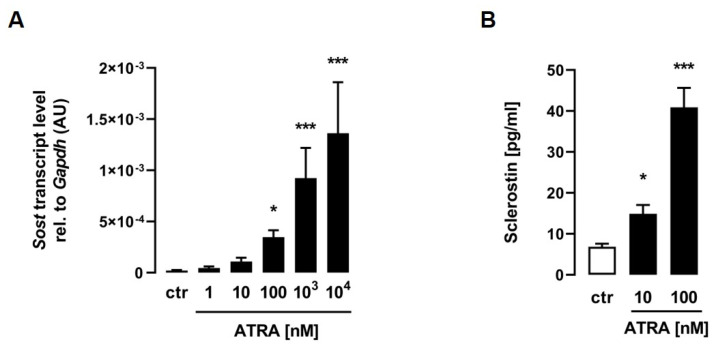
All-*trans* retinoic acid stimulates sclerostin production in C2C12 myotubes. Arithmetic means ± SEM of relative (rel.) *Sost* ((**A**) *n* = 8) mRNA abundance (normalized to *Gapdh*) or cell culture supernatant concentrations of secreted sclerostin protein ((**B**) *n* = 11) in C2C12 myotubes treated with the indicated concentrations of all-*trans* retinoic acid (ATRA) for 24 h. * *p* < 0.05 and *** *p* < 0.001 indicate significant differences from the control. AU, arbitrary units; ctr, control ((**A**,**B**) Kruskal–Wallis).

For the determination of whether ATRA induces *Sost* expression in C2C12 myotubes, differentiated cells were treated with increasing doses of ATRA for 24 h, and the mRNA abundance of *Sost* was measured by qRT–PCR. As illustrated in Figure 1A, ATRA significantly induced relative *Sost* mRNA abundance in a dose-dependent manner. To investigate whether *Sost* gene expression induced by ATRA is associated with increased sclerostin secretion, we analyzed the sclerostin protein concentrations by ELISA in the cell culture supernatant. Strikingly, the sclerostin protein secretion of C2C12 myotubes into the cell culture medium was also increased in a dose-dependent manner after 24 h of treatment with increasing concentrations of ATRA (Figure 1B). 

### 3.2. The RAR Agonist TTNPB Induces Sost Expression in C2C12 Myotubes

**Figure 2:**
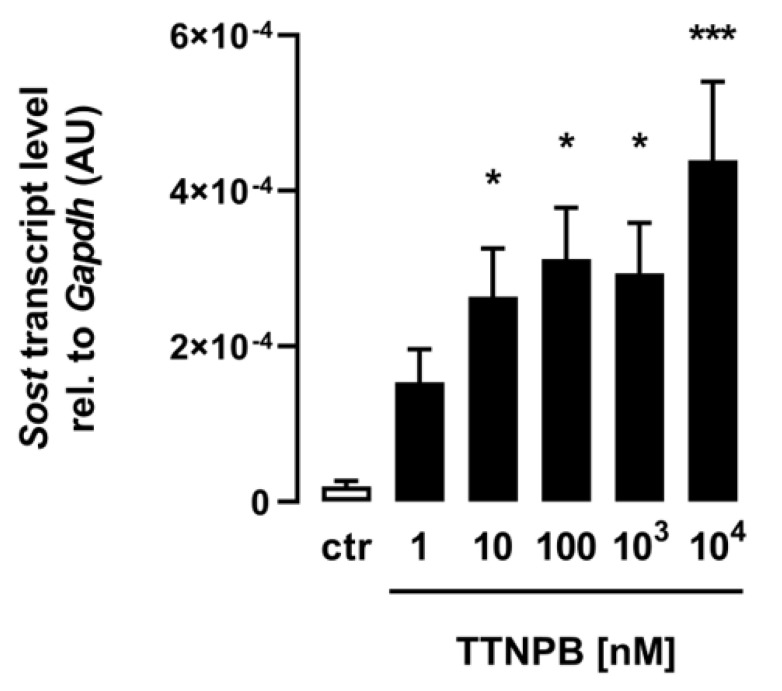
The RAR agonist TTNPB induces *Sost* expression in C2C12 myotubes. Arithmetic means ± SEM of relative (rel.) *Sost* mRNA abundance (normalized to *Gapdh*) in C2C12 myotubes (*n* = 6) treated with the indicated concentrations of the RAR agonist TTNPB for 24 h. * *p* < 0.05 and *** *p* < 0.001 indicate significant differences from the control. AU, arbitrary units; ctr, control (one-way ANOVA).

For the analysis of the effect of ATRA on *Sost* gene expression in C2C12 myotubes, the retinoic acid analog and RAR agonist TTNPB was used, and differentiated myotubes were incubated for 24 h with the same increasing concentrations used for ATRA. As shown in Figure 2, the synthetic analog TTNPB also significantly increased *Sost* gene expression in the treated C2C12 myotubes in a dose-dependent manner, corroborating the efficacy of ATRA and indicating the importance of RAR for this effect. 

### 3.3. Inhibition of RAR Impairs the Effects of Its Agonists ATRA and TTNPB in C2C12 Myotubes 

**Figure 3:**
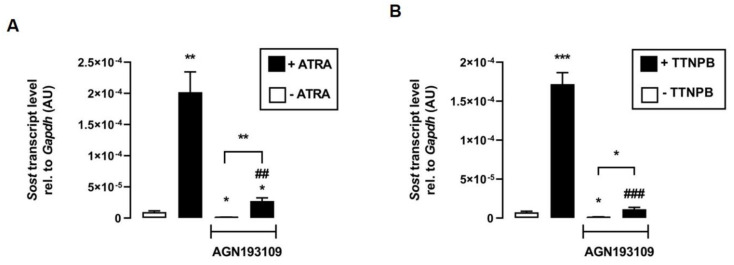
Inhibition of RAR impairs the effects of its agonists ATRA and TTNPB in C2C12 myotubes. Arithmetic means ± SEM of relative (rel.) *Sost* mRNA abundance (normalized to *Gapdh*) in C2C12 myotubes incubated without (white bars) or with (black bars) (**A**) all-*trans* retinoic acid (ATRA; 100 nM; *n* = 8) or (**B**) with RAR agonist TTNPB (100 nM; *n* = 8) in the presence or absence of the pan-RAR antagonist AGN193109 (1 µM) for 24 h. * *p* < 0.05, ** *p* < 0.01, and *** *p* < 0.001 indicate significant differences from the control. ## *p* < 0.01 and ### *p* < 0.001 indicate significant difference from the absence of the pan-RAR antagonist (2nd vs. 4th bar). AU, arbitrary units; ((**A**,**B**) Welch’s ANOVA).

The next experiments addressed the underlying mechanism of the effect of ATRA and TTNPB on *Sost* expression. Therefore, we tested whether transcriptional activation of RAR is required for the induction of *Sost* expression mediated by ATRA and its synthetic analog TTNPB. The data show that the inhibition of RAR by the pan-RAR antagonist AGN193109 significantly attenuated the stimulation of *Sost* expression by both ATRA (Figure 3A) and TTNPB (Figure 3B). Notably, ATRA and TTNPB could significantly stimulate *Sost* expression even in the presence of the RAR-inhibitor AGN193109, suggesting that both RAR ligands may also regulate relative *Sost* mRNA abundance through other signaling pathways. 

### 3.4. The Provitamin A Derivative Beta-Carotene Induces Sost Expression via Activation of RAR

**Figure 4:**
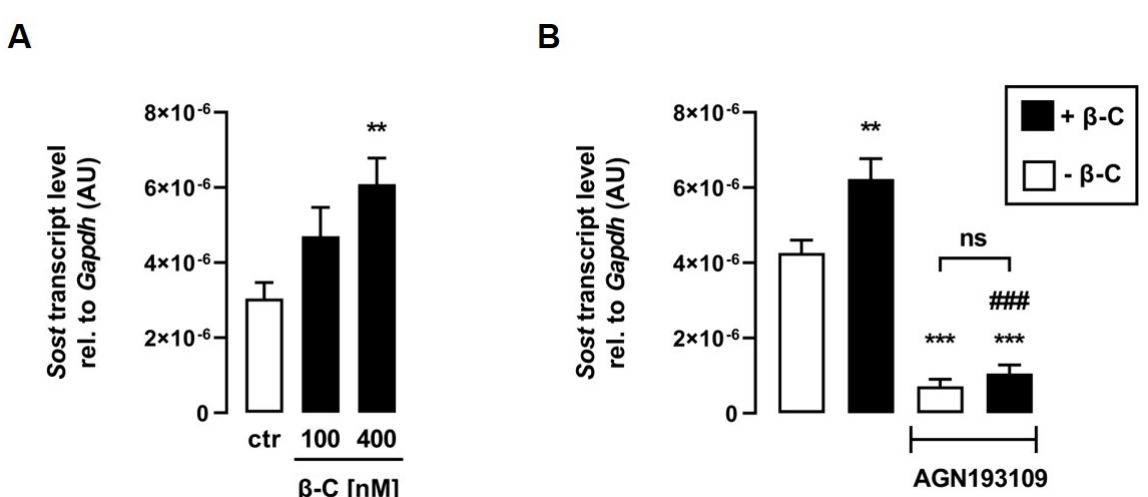
The provitamin A derivative beta-carotene induces *Sost* expression via activation of RAR. Arithmetic means ± SEM of relative (rel.) *Sost* mRNA abundance (normalized to *Gapdh*) in C2C12 myotubes incubated with (**A**) the indicated concentrations of beta-carotene (β-C; *n* = 7) or (**B**) without (white bars) or with (black bars) β-C (400 nM; *n* = 5) in the presence or absence of the pan-RAR antagonist AGN193109 (1 µM) for 24 h. ** *p* < 0.01 and *** *p* < 0.001 indicate significant differences from the control. ### *p* < 0.001 indicate significant difference from the absence of the pan-RAR antagonist (2nd vs. 4th bar). AU, arbitrary units; ctr, control; ns, not significant ((**A**,**B**) one-way ANOVA).

ATRA is synthesized from all-*trans* retinal, for which C2C12 myotubes express all necessary enzymes [28]. All-*trans* retinal, as an ATRA precursor, is also formed as a result of the cleavage of the provitamin A derivative β-C [25], which can be taken up into C2C12 cells via CD36 [37]. Thus, we investigated whether treatment with β-C also affects *Sost* transcription in C2C12 myotubes. Interestingly, β-C also increased relative *Sost* mRNA abundance in a dose-dependent manner (Figure 4A). To investigate whether the effect of β-C on *Sost* expression is also mediated by RAR, we treated C2C12 myotubes with or without β-C in the presence or absence of the RAR-inhibitor AGN193109 for 24 h. Notably, RAR inhibition significantly abolished the β-C-mediated increase in *Sost* gene expression (Figure 4B), suggesting that β-C has to be metabolized to a retinoid that can activate RAR.

### 3.5. The Effect of Beta-Carotene on Sost Transcription Depends on Bcmo1 

**Figure 5:**
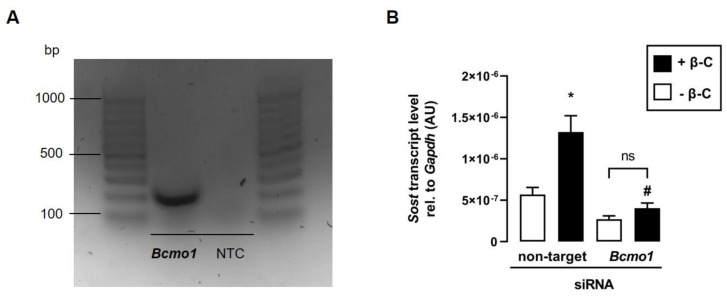
The effect of beta-carotene on *Sost* transcription depends on Bcmo1. (**A**): Original agarose gel photo showing amplified *Bcmo1*-specific cDNA in untreated murine C2C12 myotubes. (**B**): Arithmetic means ± SEM of relative (rel.) *Sost* mRNA abundance (normalized to *Gapdh*) in C2C12 myotubes incubated with nontargeting or *Bcmo1*-specific siRNA (100 nM, 120 h, *n* = 8) in the absence (white bars) or presence (black bars) of beta-carotene (β-C; 400 nM, 24 h). Treatment with *Bcmo1*-specific siRNA resulted in a 90% reduction in relative *Bcmo1* mRNA abundance. * *p* < 0.05 indicates significant differences from the control. # *p* < 0.05 indicates a significant difference from the absence of *Bcmo1*-specific siRNA (2nd vs. 4th bar). AU, arbitrary units; Bcmo1, beta-carotene 15,15′-monooxygenase 1; NTC, nontemplate control; ns, not significant ((**B**) Welch’s ANOVA).

Since BCMO1 converts β-C to all-*trans* retinal [25], which can subsequently be enzymatically metabolized to all-*trans* retinoic acid in skeletal muscle cells [26], we hypothesize that Bcmo1 activity is required for the metabolization of β-C in C2C12 myotubes to induce *Sost* transcription via the activation of RAR. As illustrated in Figure 5A, *Bcmo1* is expressed in C2C12 myotubes, suggesting that β-C can be converted to all-*trans* retinal and subsequently to biologically active ATRA. To determine whether the cleavage of β-C is necessary for the induction of *Sost* transcription, we transfected C2C12 myoblasts with *Bcmo1*-specific siRNA. As hypothesized, the β-C-mediated increase in *Sost* transcription vanished in the presence of *Bcmo1*-specific siRNA (Figure 5B).

## 4. Discussion

This study provides evidence that the biologically active vitamin A metabolite ATRA and provitamin A β-C are potent regulators of sclerostin expression in muscle cells. The data demonstrate that ATRA-induced sclerostin synthesis is also associated with a corresponding cellular release of sclerostin protein, suggesting that muscle cells may also contribute to the endocrine sclerostin effect after stimulation with vitamin A. In this context, an ATRA concentration of 10 nM, which is comparable to plasma concentrations found in healthy individuals [38,39], led to a significant increase in sclerostin secretion. Our results support the observation of two studies that also observe a stimulatory effect of ATRA on *Sost* gene expression [34,40]. In contrast, Lind et al. observed an inhibitory effect of ATRA on *Sost* gene expression in bone cells [41]. Although bone is the major cellular source of sclerostin, our results show ATRA-mediated stimulation of sclerostin formation outside bone, as a classic sclerostin-forming tissue, suggesting that ATRA may function as a general regulator of sclerostin formation. Recent studies have shown that in addition to bone and muscle, the *SOST* gene is also present in the liver [42], kidney [17], and arteries [43], suggesting that ATRA may also regulate sclerostin formation in these tissues and thus potentially affect bone homeostasis or mediate non-skeletal effects.

Our experiments also investigated the molecular mechanisms through which ATRA exerts its stimulatory effect on sclerostin. Because RAR is the classic transcription factor to which ATRA binds to mediate its genomic effects [22], it was hypothesized that RAR might also be central to ATRA-mediated *Sost* gene expression in the muscle. According to our hypothesis, RAR is identified as the important transcription factor that mediates the effect of ATRA on *Sost* because ATRA-induced *Sost* expression was reduced, although not abolished, in RAR-inhibited C2C12 myotubes. However, there are different isoforms of RAR, the RAR-α, -β, or -γ [22]. The RAR-inhibitor AGN193109 which was used in our experiment acts as a pan-RAR antagonist, and ATRA and TTNPB function as pan-RAR agonists. Thus, it was not possible to identify the relevant RAR isoform that is mainly responsible for the *Sost* gene expression in C2C12 myotubes. However, it is reasonable to speculate that RAR-γ is the most relevant transcription factor, as it shows the highest expression level in C2C12 myotubes among the RAR isoforms, and the stimulatory effect of ATRA is abolished in C2C12 myotubes with silenced RAR-γ [44]. The remaining *Sost* expression even in the presence of the inhibited RAR suggests that ATRA can also induce *Sost* gene expression via other RAR-independent signaling pathways. Interestingly, data from other studies demonstrate that the treatment of C2C12 cells with ATRA leads to activation of 5′ AMP-activated protein kinase (AMPK) [35,45], which is a known stimulator of *Sost* gene expression in bone [46]. Therefore, AMPK might also contribute to the observed *Sost* gene expression following ATRA treatment. 

Additionally, our data show that C2C12 myotubes can express *Bcmo1*, which is consistent with data from muscular cells of nonmurine origin [26]. This finding suggests that muscle cells are capable of metabolizing the provitamin A derivative β-C to all-*trans* retinal, which is confirmed by the observation that β-C, similar to ATRA, could stimulate *Sost* expression in C2C12 myotubes via the activation of RAR. Interestingly, β-C does not have an ATRA-independent effect because in the presence of siRNA-mediated *Bcmo1* knockdown, the stimulatory effect of β-C on the relative *Sost* mRNA abundance vanished. This result shows that β-C must first be metabolized to all-*trans* retinal by Bcmo1 and then metabolized to ATRA to induce stimulation of *Sost* gene expression. 

The vitamin A metabolite ATRA affects bone and muscle tissue homeostasis [22,27,28,29,30,31], and interestingly, increasing evidence has identified a functional role in muscle–bone crosstalk through the regulation of myokine and osteokine production [32,33,34,35]. Our data support this regulatory importance of ATRA and, interestingly, of its precursor β-C for the release of myokines through the identification of the downstream target sclerostin. In this study, we identified ATRA as a potent stimulator of muscular formation and secretion of sclerostin, an important antagonist of bone growth [15]. This result is consistent with observations that high vitaminA intake in rodents results in thinner bones and reduced bone formation, growth, and mineralization compared with those of the controls [41,47,48]. The RAR isoform that is responsible for the poorer bone morphology has not been fully elucidated [22]. However, data indicate that the resorptive effects of ATRA on bone are predominantly mediated via RAR-α [49]. In addition, increased retinol intake in humans is associated with the development of osteoporosis and increased risk of fractures [27]. Since sclerostin is an important regulator of bone homoeostasis [17] and an independent risk factor for osteoporosis-related fractures [50,51], it is reasonable to speculate that the poorer bone health and the increase in fracture risk with excessive vitamin A intake observed in these studies may also be a consequence of increased muscular sclerostin formation. This hypothesis is supported by recent in vivo data demonstrating an inhibitory effect of muscle sclerostin on adjacent bone [13]. However, an adequate intake of vitamin A from food and supplements maintains bone homeostasis, and β-C may also have protective effects on bone [27], in contrast to our results. However, excessive β-C intake results in lower efficiency of conversion to a retinoid because high ATRA concentrations decrease *BCMO1* expression, thus preventing excess retinoid and ATRA production via BCMO1 [52]. However, since our β-C concentrations used are within the physiological range [53], it can be speculated that the protective effects of β-C on bone are mediated independently of the effect on sclerostin formation. Interestingly, ATRA is used to treat patients with acute promyelocytic leukemia, a subtype of acute myeloid leukemia [54], which is associated with bone pain in these patients [55]. Based on the present data, it would therefore be interesting to measure circulating levels of sclerostin and bone mass in patients with acute promyelocytic leukemia treated with ATRA. 

Moreover, whether ATRA-mediated sclerostin formation mediates possible paracrine effects on the surrounding musculature is an open question. Because muscular *SOST* expression is higher in patients with multiple myeloma and sarcopenia than in control subjects [56] and circulating sclerostin levels are negatively correlated with skeletal muscle mass [57], it is tempting to speculate that sclerostin may have an inhibitory effect on muscle cell proliferation and differentiation by impairing balanced canonical Wnt/β-catenin signaling which regulates skeletal muscle regeneration and myogenesis [58]. However, the role of sclerostin as a possible inhibitor of myogenesis remains unclear. In contrast, β-C has been shown to increase muscle mass and hypertrophy in mice [59], which contradicts the hypothesis of an inhibitory effect of sclerostin on myogenesis, at least in view of our data and the β-C-mediated stimulation of *Sost*.

Since it has already been demonstrated that dietary vitamin A intake is related to the concentrations of other circulating myokines, such as myostatin [60], our data suggest that sclerostin formation may also be regulated by dietary vitamin A and carotenoid intake. This finding provides a basis for further studies that investigate the influence of dietary factors and nutrients on sclerostin formation affecting the homeostasis of other adjacent tissues.

Taken together, this study is the first to show a stimulatory effect of ATRA and β-C on sclerostin synthesis and release in C2C12 myotubes by activating RAR. However, the β-C effect on *Sost* gene expression depends on the conversion of β-C to all-*trans* retinal by Bcmo1. These results provide evidence for the regulatory importance of vitamin A derivatives for muscle–bone crosstalk via the release of sclerostin with antianabolic effects on bone and provide further insight into the complex myokine-mediated homeostasis between muscle and bone.

## Data Availability

The data presented in this study are available on request from the corresponding author.
